# The effect of lumbar medial branch radiofrequency neurotomy on cobb angle progression in individuals with adult scoliosis compared to natural history: A cross-sectional study

**DOI:** 10.1016/j.inpm.2024.100411

**Published:** 2024-04-17

**Authors:** Marc Caragea, Austin Le, Tim Curtis, Amelia Ni, Tyler Clark, Andrew Joyce, Colton Hickman, Brandon Lawrence, Zane Randell, Perry Goodman, Addisyn Poduska, Michella Rasmussen, Amanda Cooper, Masaru Teramoto, Allison Glinka Przybysz, Taylor Burnham, Aaron Conger, Zachary L. McCormick

**Affiliations:** a Department of Physical Medicine and Rehabilitation, University of Utah School of Medicine, Salt Lake City, UT, USA; b Department of Orthopedics, University of Utah School of Medicine, Salt Lake City, UT, USA; c School of Medicine, University of Utah, Salt Lake City, UT, USA; d Department of Orthopedics, Division of Physical Medicine and Rehabilitation, Washington University in St. Louis, St. Louis, MO, USA

**Keywords:** Facet, Ablation, Instability, Multifidi, Spinal

## Abstract

**Background:**

Lumbar radiofrequency neurotomy (LRFN) effectively alleviates zygapophyseal joint-mediated pain by coagulating medial branch nerves to disrupt nociceptive signaling pathways. The concomitant denervation of multifidus fibers has led to concern that LRFN may increase segmental instability and accelerate degenerative changes in patients with certain pre-existing spinal pathologies. There is a paucity of literature evaluating whether LRFN increases the progression of spinal curvature in patients with adult scoliosis.

**Objective:**

Compare the lumbosacral Cobb angle progression rate in patients with adult scoliosis who underwent LRFN to the annual progression rate of 0.83 ± 1.1° expected by natural history.

**Design:**

Cross-sectional study.

**Methods:**

Consecutive patients diagnosed with adult scoliosis who underwent LRFN to treat zygapophyseal joint-related low back pain were identified. Patient demographics, LRFN procedure details, and radiographs confirming scoliosis were collected from electronic medical records. Pre- and post-LRFN radiographs were used to calculate the average annual rate of Cobb angle progression. Data were analyzed using a Wilcoxon signed-rank test and a linear regression model.

**Results:**

Sixty patients (mean age 69.2 ± 11.6 years; 70.0 % female) met the criteria and were included in the analyses. The mean time to radiographic follow-up was 35.0 ± 22.7 months post-LRFN. The average Cobb angle progression was 0.54 ± 3.03° per year and did not differ significantly from the known natural progression rate of 0.83 ± 1.1° per year. None of the included covariates (body mass index, LRFN laterality, and number of levels denervated) were significantly associated with the average annual Cobb angle progression rate.

**Conclusions:**

Our results suggest that LRFN has no appreciable effect on the rate of Cobb angle progression in patients with adult scoliosis.

## 1 Introduction

Adult scoliosis is a spinal deformity in skeletally mature individuals characterized by the presence of an abnormal curve in the coronal plane measuring greater than 10°, according to the Cobb method [1]. The prevalence of adult scoliosis varies widely in the literature, estimated between 6 % and 68 %, and is thought to increase with age [[2], [3], [4]]. Given the demographic shift towards an aging population in the United States, adult scoliosis is a growing clinical concern, heightened by its associated negative impact on health-related quality of life [5,6].

Adult scoliosis can be further categorized into idiopathic and de novo types. Adult idiopathic scoliosis (AIS) refers to a history of adolescent idiopathic scoliosis with progression of deformity or increasing symptoms in adulthood [7]. In adult de novo or adult degenerative scoliosis (ADS), curvature develops in adulthood due to deterioration of spinal motion segments with age, typically beginning with asymmetrical degenerative changes to the intervertebral disc that leads to subsequent deterioration of posterior elements, particularly the zygapophyseal “facet” joints [7,8]. While the true prevalence of zygapophyseal joint pain in the ADS population is unknown, the zygapophyseal joints have been implicated as a common pain generator in patients with ADS and axial pain symptoms [7].

Lumbar medial branch radiofrequency neurotomy (LRFN) is commonly used to treat zygapophyseal joint-mediated pain, including in patients with spinal deformities such as scoliosis [10]. When appropriate patient selection and procedural techniques are applied, LRFN is a well-established, safe, and effective treatment [9,[11], [12], [13], [14], [15], [16], [17], [18]]. Electrodiagnostic data has validated the procedure, demonstrating that coagulation of targeted medial branch nerves results in segmental denervation of the associated multifidus muscle [9,19]. However, this raises a theoretical concern that denervation and consequent atrophy of multifidi muscles, essential spine stabilizers, could lead to segmental instability, accelerated degeneration, and exacerbation of scoliosis [20]. While some degree of post-procedural atrophy of the multifidi muscles has been reported, the clinical implications of this potential complication are not well understood [19,20]. LRFN has not been shown to influence zygapophyseal joint degeneration [19]. Despite this, some clinicians may not consider patients with spinal deformities, such as scoliosis, appropriate candidates for LRFN due to concerns of accelerating the rate of deformity progression. It is unknown whether multifidus muscle denervation due to LRFN leads to acceleration of curvature progression beyond that expected by natural history [20]. In individuals with adult scoliosis, the Cobb angle has been described to increase at a rate of 0.83 ± 1.1° per year without surgical intervention, with more rapid progression observed in those with ADS compared with AIS [21].

This study aims to investigate the relationship between LRFN and Cobb angle progression in adults with scoliosis, comparing Cobb angle progression in patients with degenerative scoliosis who underwent LRFN to expected progression based on natural history.

## 2 Methods

### 2.1 Data collection

This single-center, retrospective cross-sectional study was approved by the University of Utah Institutional Review Board (IRB 138414). The electronic medical records of consecutive patients who underwent LRFN from July 2014–May 2020 were identified and reviewed through an electronic database search of the CPT code for LRFN (64635). The inclusion criteria for patients were 1) ≥18 years of age with pre-existing lumbosacral scoliosis (Cobb angle >10°), 2) LRFN for the treatment of zygapophyseal joint-mediated low back pain as confirmed by dual, concordant diagnostic medial branch blocks, 3) failure of at least six weeks of conservative treatments (such as physical therapy, activity modification, and oral analgesics), 4) baseline neutral weight-bearing anterior-posterior radiographs of the lumbar spine displaying scoliosis taken within the year before LRFN, and 5) corresponding X-rays obtained within 6 months–10 years post-LRFN to evaluate the degree of scoliosis progression. Patients were excluded if they were under 18 years old, lacked pre- and/or post-procedure radiographs, or had pars defect(s) noted on imaging.

### 2.2 Radiographic evaluation

Pre- and post-procedural posterior-anterior scoliosis radiographs were utilized to calculate the Cobb angle in the coronal plane using standard, computer-assisted techniques [22]. Baseline, pre-procedural radiographs were obtained within one year pre-LRFN and post-procedural radiographs from six months to ten years post-LRFN. Cobb angle measurements were performed by three study authors (MC, TC, and CH). Two individuals independently reviewed each radiograph, with any disagreements resolved by a third reviewer. The annual rate of Cobb angle progression was calculated utilizing the following formula:

(Post-RFN Cobb angle degree – Pre-RFN Cobb angle degree) / (12 Months/Months Follow-up)

### 2.3 Procedures

All LRFN procedures were performed by physician specialists in Physical Medicine and Rehabilitation who had received fellowship training in either Pain Medicine, Sports Medicine, or Interventional Spine and Musculoskeletal Medicine.

### 2.4 Statistical analysis

The primary outcome measure was the yearly Cobb angle progression rate. Descriptive statistics (mean and standard deviation for continuous variables; frequency and percentage for categorical variables), and confidence intervals (CIs) were calculated for patient demographic and clinical variables. A one-sample Wilcoxon signed-rank test was used to compare the annual Cobb angle progression rate with the reference value of 0.83°. Further, linear regression analysis was performed to explore whether select demographic and clinical covariates were significantly correlated with the annual Cobb angle progression rate. Standard errors and CIs of the coefficients were computed using the bootstrap estimation with 1000 replications [[23], [24], [25]]. The threshold value for statistical significance was set at *p* < 0.05. All the analyses were performed using Stata/MP 18.0 (StataCorp LLC, College Station, TX).

## 3 Results

**Fig. 1:**
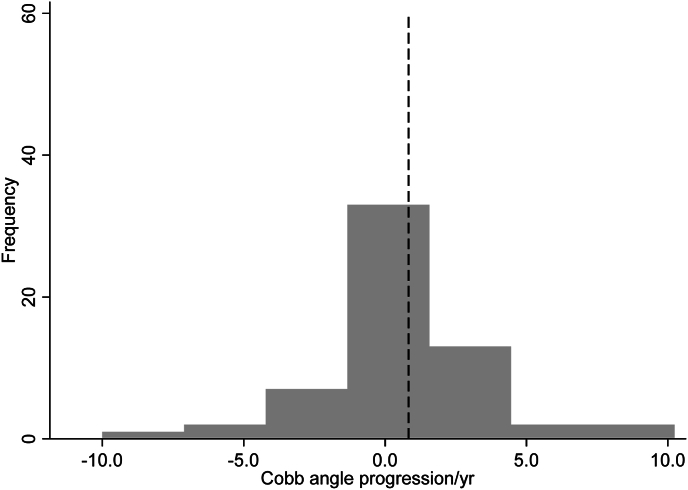
Histogram of annual Cobb angle progression. Note: Dashed line represents 0.83°, the reference value for annual Cobb angle progression.

A total of 60 patients were included in the analysis (mean age 69.2 ± 11.6 years; 70.0 % female). The mean time from LRFN treatment to follow-up radiographic assessment was 35.0 ± 22.7 months. In this cohort, initial Cobb angles ranged from 10 to 44°. Patient demographics are presented in Table 1a and 1b. On average, Cobb angle progressed by 0.54 ± 3.03° per year. There was no statistically significant difference in mean annual Cobb angle progression post-LRFN compared to the expected natural progression rate of 0.83° per year (95 % CI: 0.24, 1.32; *p* = 0.272; Table 2 and Fig. 1). The linear regression model indicated that BMI, LRFN laterality, and number of levels denervated were not significantly associated with annual Cobb angle progression (*p* > 0.05; Table 3).

**Table 1a:** Patient demographics, clinical, and procedure-related variables (*N* = 60; quantitative variables).

Variable	*N*	Mean	SD	Min	Max
Age	60	69.2	11.6	29.0	91.0
BMI (kg/m^2^)	60	29.3	7.4	18.8	50.4
Follow-up months	60	35.0	22.7	7.0	113.0
Pelvic incidence (initial)	53	52.7	15.1	16.0	87.0
Pelvic incidence (follow-up)	50	52.1	15.7	11.0	79.0
Pelvic tilt (initial)	53	23.3	12.2	1.0	68.0
Pelvic tilt (follow-up)	50	22.6	10.5	1.0	45.0

**Table 1b:** Patient demographics, clinical, and procedure-related variables (*N* = 60; categorical variables).

Variable	No.	%
Gender		
Male	18	30.0
Female	42	70.0
Ethnicity		
Not Hispanic/Latino	58	96.7
Hispanic/Latino	2	3.3
Concomitant scoliosis		
Yes	60	100.0
No	0	0.0
Spinal surgery after initial LRFN		
Yes	16	26.7
No	44	73.3
Spinal fusion after LRFN		
Yes	11	18.3
No	49	81.7
LRFN laterality		
Left	13	21.7
Right	12	20.0
Bilateral	35	58.3
LRFN laterality		
Unilateral	25	41.7
Bilateral	35	58.3
Number of levels denervated		
One	17	28.3
Two	40	66.7
Three	3	5.0
Laterality of scoliosis convexity		
Left	34	56.7
Right	26	43.3
History of lumbar surgery prior to LRFN		
Yes	10	16.7
No	50	83.3
History of spinal fusion prior to LRFN		
Yes	8	13.3
No	52	86.7

LRFN = lumbar radiofrequency neurotomy.

**Table 2:** Cobb angle annual progression rate.

Variable	*n*	Mean (SD)	Median (IQR)	Min, Max	95 % CI	*p*^a^
Cobb angle progression (degrees/year)	60	0.54 (3.03)	0.35 (−0.86, 1.97)	−10.00, 10.24	−0.24, 1.32	0.272

a From significance test (vs. 0.83°/year), using one-sample Wilcoxon signed-rank test.

**Table 3:** Linear regression model on annual Cobb angle progression by select covariates.

Covariate	B	95 % CI^a^	*p*
Body mass index	−0.03	−0.15, 0.08	0.658
RFA laterality (vs. unilateral)			
Bilateral	0.49	−1.03, 1.98	0.519
Number of levels denervated (vs. one)			
Two	0.28	−1.44, 1.87	0.739
Three	−1.57	−3.61, 0.59	0.127

B = beta coefficient; CI = confidence interval; RFA = radiofrequency ablation.

Note: Outcome = annual Cobb angle progression.

a Calculated from bootstrap standard errors with 1000 replications.

## 4 Discussion

Here we report the average lumbosacral Cobb angle progression rate in patients with adult scoliosis who received LRFN and compare it to the described natural progression rate of 0.83 ± 1.1° per year [21]. Despite the theoretical risk of spinal destabilization as an adverse effect of LRFN, we found no statistically significant difference in the average annual rate of scoliosis progression for the present study cohort (0.54 ± 3.03° per year) and the reference value of 0.83°. The results of this study suggest that LRFN at a limited number of contiguous levels can be used to treat zygapophyseal joint-mediated pain in adult scoliosis patients. Importantly, this treatment approach does not have a clinically meaningful impact on the rate of curve angle progression. This finding is consistent with prior reports that LRFN does not result in the progression of spondylolisthesis at a rate greater than that expected by natural history alone [26,27].

The effect of LRFN on the progression of scoliosis has been a topic of interest due to the potential for denervation and subsequent atrophy of stabilizing lumbar multifidi muscles that could hypothetically worsen the curve angle progression of scoliosis. Forming the posterior portion of the inner muscular ring of the spine, the multifidus is a series of postural support muscles that contribute to the stability of vertebral motion segments; as such, atrophy of multifidi muscle sections secondary to disruption of motor inputs could lead to spinal destabilization [28]. Similarly, the longissimus, iliocostalis, and spinalis muscles may also be at risk of denervation if the dorsal ramus' intermediate and/or lateral branches are inadvertently damaged during LRFN [29].

The natural progression of adult scoliosis has been reported in the literature. Initial descriptions of curvature progression rate were limited to individuals with idiopathic adult scoliosis. Ohashi et al. performed a long-term, retrospective study of 147 patients with AIS and found that the Cobb angle at the thoracolumbar-lumbar curve increased from a mean of 37.3–47.8° at a rate of 0.41 ± 0.39° per year [30]. The authors concluded that L3 tilt beyond 16° at skeletal maturity independently predicted scoliosis curve progression beyond 0.5° per year. Watanabe et al. published results from another long-term retrospective cohort study of AIS patients and noted a mean thoracolumbar-lumbar curve progression of 7.2° over 23.5 years, corresponding to an annual Cobb angle progression rate of 0.3° [31]. The investigators also identified an association between decreased skeletal muscle volume and fatty degeneration of the lumbar extensor muscles in those with major curvature beyond 30° in adulthood. In 2020, Faraj et al. characterized curvature progression rates in a retrospective cohort of 58 non-surgical patients with degenerative and idiopathic forms of adult scoliosis over a mean of 59.8 ± 34.5 months. The investigators found that the average Cobb angle increased at a rate of 0.83 ± 1.1° per year, with more rapid progression observed in those with ADS compared with AIS [21].

### 4.1 Limitations

The results of our study should be interpreted within the context of data collection from a single institution, which may be different from other clinical practices or patient demographics. These findings reflect the specific circumstances and characteristics of the institution and may not be generalizable to other settings. Furthermore, the retrospective study design and small sample size also pose inherent limitations that should be considered when interpreting the results.

Because LRFN is rarely performed for adolescents (and long-term historical x-rays were not consistently available), we could not stratify our study population according to idiopathic and degenerative subtypes. This distinction is particularly important when considering that the clinical course of adult scoliosis almost certainly depends on etiology [21], suggesting LRFN could influence curvature progression differently in patients diagnosed with AIS versus ADS. Cobb angle measurements themselves are error prone due to 1) variable production of spinal radiographs and 2) inter-observer variability, 3) wrong definitions of the end vertebrae, and 4) defective angle measurements with total differences of less than ±5° not reaching clinical significance in past studies [32,33].

Patient eligibility criteria for the present study were more relaxed than previous work examining adult scoliosis progression rates. While a prior history of surgery was listed among the exclusion criteria of earlier investigations [21,30,31], a small subset of our study cohort was comprised of patients who had previously undergone spinal surgeries, including fusions. Faraj et al. restricted their analysis to patients greater than 40 years of age with baseline and follow-up radiographs taken at least 24 months apart [21]. In contrast, the present study included patients over 18, and follow-up radiograph timing ranged from 6 to 113 months. Notably, this does improve the generalizability of our findings.

The reference Cobb angle progression rate of 0.83° per year used in our study was obtained from a retrospective analysis by Faraj et al. of non-surgical patients with idiopathic and degenerative forms of adult scoliosis [21]. Further research is necessary to fully validate this reference rate. As stated above, there are other studies [30,31] detailing the rates of progression in AIS patients, but there is no standardized set of inclusion/exclusion criteria, making direct comparisons difficult. A larger cohort and a more extended follow-up period are required to characterize the effects of LRFN on scoliosis progression more definitively. Additionally, there may be a nonlinear progression that should be considered with a longer time frame. These findings emphasize the importance of additional research to gain a deeper understanding of the progression of scoliosis and potential predictive factors.

## 5 Conclusion

Given the high prevalence of concomitant adult scoliosis and zygapophyseal joint pain, it is essential to consider the potential consequences of treating zygapophyseal joint pain with techniques such as LRFN on Cobb angle progression. In this cross-sectional study, lumbosacral scoliosis did not progress at a rate greater than expected by natural history following LRFN at a limited number of contiguous levels. Further investigation is warranted to more definitively characterize the expected natural rate of Cobb angle progression in patients with adult scoliosis, and more longitudinal studies with larger cohorts could help better establish the effects of LRFN on scoliosis progression.

## Funding

This study was funded by research grants from (1) the Skaggs Foundation for Research and (2) the Mitchell and June Morris Foundation.

## Declaration of competing interest

The authors declare the following financial interests/personal relationships which may be considered as potential competing interests: Zachary McCormick reports financial support was provided by Skaggs Foundation for Research. Zachary McCormick reports financial support was provided by Mitchell and June Morris Foundation. Zachary McCormick reports a relationship with International Pain and Spine Intervention Society that includes: board membership. Zachary McCormick reports a relationship with Avanos Medical Inc that includes: funding grants. Taylor Burnham reports a relationship with Avanos Medical Inc that includes: consulting or advisory. Taylor Burnham reports a relationship with Diros Technology Inc that includes: funding grants. Aaron Conger reports a relationship with Stratus that includes: funding grants. If there are other authors, they declare that they have no known competing financial interests or personal relationships that could have appeared to influence the work reported in this paper.
